# *Salmonella* *enterica* serovar Typhimurium BaeSR two-component system positively regulates *sodA* in response to ciprofloxacin

**DOI:** 10.1099/mic.0.066787-0

**Published:** 2013-10

**Authors:** P. Guerrero, B. Collao, R. Álvarez, H. Salinas, E. H. Morales, I. L. Calderón, C. P. Saavedra, F. Gil

**Affiliations:** 1 Laboratorio de Microbiología Molecular, Departamento de Ciencias Biológicas, Facultad de Ciencias Biológicas, Universidad Andres Bello, Santiago, Chile; 2 Department of Biomolecular Chemistry, University of Wisconsin-Madison, USA; 3 DOE Great Lakes Bioenergy Research Center, University of Wisconsin-Madison, USA

## Abstract

In response to antibiotics, bacteria activate regulatory systems that control the expression of genes that participate in detoxifying these compounds, like multidrug efflux systems. We previously demonstrated that the BaeSR two-component system from *Salmonella enterica* serovar Typhimurium (*S*. Typhimurium) participates in the detection of ciprofloxacin, a bactericidal antibiotic, and in the positive regulation of *mdtA*, an efflux pump implicated in antibiotic resistance. In the present work, we provide further evidence for a role of the *S*. Typhimurium BaeSR two-component system in response to ciprofloxacin treatment and show that it regulates *sodA* expression. We demonstrate that, in the absence of BaeSR, the transcript levels of *sodA* and the activity of its gene product are lower. Using electrophoretic mobility shift assays and transcriptional fusions, we demonstrate that BaeR regulates *sodA* by a direct interaction with the promoter region.

## Introduction

Current antimicrobial therapies, which cover a wide array of targets, fall into two general categories: bactericidal drugs, which kill bacteria with an efficiency of >99.9 %, and bacteriostatic drugs, which inhibit growth (Pankey & Sabath, 2004). Antibacterial drug target interactions are well studied and fall into three classes: inhibition of DNA replication and repair, inhibition of protein synthesis, and inhibition of cell-wall turnover (Walsh, 2000).

In the search for new antibiotic targets, two-component systems (TCSs) have emerged as extremely attractive ones. In prokaryotes, TCSs transduce and detect signals such as pH, temperature, osmolarity, light, nutrients, ions and toxins, regulating a wide range of processes including motility, virulence, metabolism, developmental switches, antibiotic resistance and stress responses (Batchelor & Goulian, 2003; Cai & Inouye, 2002; Stock *et al.*, 2000). TCSs are widespread in bacteria and, so far as is known, absent in mammals (Stock *et al.*, 2000); therefore, they could be used as targets for antibiotics. Perhaps one of the most compelling reasons to target TCSs is that they are used by pathogenic bacteria to control the expression of virulence factors, which are required for a successful infective cycle (Stock *et al.*, 2000).

In *Escherichia* *coli*, BaeSR were first described as putative members of a TCS (Nagasawa *et al.*, 1993) involved in drug resistance by regulating the expression of genes that encode drug transporters (Baranova & Nikaido, 2002; Nagakubo *et al.*, 2002), and participating in the response to envelope stress (Leblanc *et al.*, 2011). Supporting a role for BaeSR in drug resistance, overexpression of BaeR significantly increases the resistance of an *E. coli acrAB* mutant to novobiocin and deoxycholate, by upregulating a multiple drug transport system encoded by the *mdtABCD* operon (Baranova & Nikaido, 2002; Nagakubo *et al.*, 2002; Nishino *et al.*, 2007). In *Salmonella enterica* serovar Typhimurium (*S*. Typhimurium), a BaeR mutant is fourfold more sensitive to ceftriaxone (Hu *et al.*, 2005), and the BaeSR TCS responds to the fluoroquinolone ciprofloxacin (CIP) (Guerrero *et al.*, 2012), which contributes to bacterial cell death by inhibiting DNA gyrase (Cambau & Gutmann, 1993). Fluoroquinolones have been successfully used to treat salmonellosis, are commonly the first choice for treatment, and have also proven useful for the treatment of infections caused by multiple antibiotic-resistant strains (Piddock, 2002).

Understanding the mechanisms by which antibiotics act is very important to improve current therapies. For several years, a model for antibiotic killing that was independent of their mode of action was well accepted (Kohanski *et al.*, 2007). The mechanism involved formation of reactive oxygen species (ROS) due to antibiotic exposure, as a result of hyperactivation of the electron transport chain, which in turned killed bacteria. However, recent studies in *E. coli* have challenged this model and shown that antibiotic treatment does not accelerate hydrogen peroxide generation or elevate the levels of free intracellular iron, an essential reactant for the production of ROS-induced damage (Liu & Imlay, 2013; Keren *et al.*, 2013).

Despite the recent findings that antibiotics kill through a ROS-independent mechanism (Liu & Imlay, 2013; Keren *et al.*, 2013), studies in *E*. *coli* show that the expression of genes that code the superoxide dismutases (SODs) SodA and SodB are induced by ofloxacin (Kaldalu *et al.*, 2004) and SodA by CIP (Smirnova *et al.*, 2012). In the present study, we investigated the role of the BaeSR TCS in the regulation of *sodA* and *sodB* in response to CIP. First, we show that in a Δ*baeSR* strain there is a decrease in total SOD activity, specifically in that of SodA. This decrease in activity correlated with lower transcript levels of *sodA* in the *baeSR* mutant strain, as measured by real-time RT-PCR (qRT-PCR) and using transcriptional fusions. Bioinformatic analysis predicted the presence of two BaeR-binding sites (BBSs) at the *sodA* promoter region. Using electrophoretic mobility shift assays (EMSAs), transcriptional fusions, and mutation of the *sodA* promoter region, we show that BaeR exerts its regulation by direct interaction with one of the predicted sites. Interestingly, the binding site required for the positive regulation of *sodA* in response to CIP is not required for the regulation in response to menadione (MEN), suggesting different mechanisms of regulation.

## Methods

### 

#### Bacterial strains and growth conditions.

*Salmonella* strains used in this study are listed in Table 1. Bacteria were grown routinely at 37 °C in Luria–Bertani broth (LB) with shaking. When required, LB was supplemented with ampicillin (Amp, 100 mg l^−1^). Solid media included 15 g agar l^−1^. When necessary, growth medium was treated with CIP (0.91 µM) or MEN (50 µM).

**Table 1.** Strains used in this study

Strain	Relevant characteristic(s) or genotype	Source
***S.* Typhimurium**		
14028s	Wild-type strain	G. Mora*
Δ*baeSR*	*baeSR* : : FRT	This work
14028s/p*sodA*-*lacZ*	Wild-type strain with pLacZ vector carrying 600 bp of *sodA* promoter	This work
14028s/pMUTA-*lacZ*	Wild-type strain with pLacZ vector carrying *sodA* promoter with mutated BBS1 box	This work
14028s/pMUTB-*lacZ*	Wild-type strain with pLacZ vector carrying *sodA* promoter with mutated BBS2 box	This work
14028s/pMUTAB-*lacZ*	Wild-type strain with pLacZ vector carrying *sodA* promoter with mutated BBS1 and BBS2 boxes	This work
Δ*baeSR*/p600*sodA*-*lacZ*	Δ*baeSR* strain with pLacZ vector carrying 600 bp of *sodA* promoter	This work
Δ*baeSR*/pMUTA-*lacZ*	Δ*baeSR* strain with pLacZ vector carrying *sodA* promoter with mutated BBS1 box	This work
Δ*baeSR*/pMUTB-*lacZ*	Δ*baeSR* strain with pLacZ vector carrying *sodA* promoter with mutated BBS2 box	This work
Δ*baeSR*/pMUTAB-*lacZ*	Δ*baeSR* strain with pLacZ vector carrying *sodA* promoter with mutated BBS1 and BBS2 boxes	This work
***E. coli***		
BL21(DE3)	F^−^ *ompT gal dcm lon hsdS*_B_(r_B_^−^ m_B_^−^) λ(DE3) (*lacI lacUV5-T7* gene 1 *ind1 sam7 nin5*)	Invitrogen
BL21/pET-TOPObaeR	BL21(DE3) transformed with the pET-TOPO-*baeR* vector carrying the *S*. Typhimurium *baeR* gene	Guerrero *et al.* (2012)

* G. Mora, Laboratorio de Microbiología, Universidad Andres Bello.

#### Determination of SOD activity.

Ten millilitres of bacterial cultures grown to OD_600_ ~0.4 were exposed to CIP or MEN for 10 min. SOD activity was assessed by measuring the inhibition of the photochemical reduction of nitro blue tretrazolium (NBT) from crude extracts as described elsewhere (Jakubowski *et al.*, 2000). The reaction mixture (1 ml) contained 50 mM potassium phosphate buffer (pH 8.5), 0.1 mM EDTA, 0.02 mM riboflavin, 13 mM methionine, 0.6 mM NBT and the crude protein extract (45 µg). *A*_550_ was measured after 15 min illumination. A SOD unit was defined as the amount of enzyme causing 50 % inhibition of NBT reduction. For in-gel SOD activity, electrophoresis was carried out at 4 °C in 10 % polyacrylamide mini-slab gels in standard Tris-glycine buffer (pH 8.3), loading 10 µg each crude extract. After electrophoresis, the photochemical method described by Beauchamp & Fridovich (1971) was modified and used to determine SOD activity on gels. Briefly, the gel was soaked in 50 mM potassium phosphate buffer (pH 8.5), 0.1 mM EDTA, 0.02 mM riboflavin, 13 mM methionine, 0.6 mM NBT. The gel was illuminated with a light intensity of 30 mE m^−2^ s^−1^ for 15 min to initiate the photochemical reaction. All the procedures were carried out at room temperature.

#### RNA isolation and mRNA detection.

An overnight bacterial culture (wild-type and TCS mutant strains) was diluted 100-fold with fresh LB medium and grown at 37 °C, with shaking, up to OD_600_ ~0.4. The culture was split into two 10 ml aliquots and one of them was incubated with CIP or MEN. Cells were grown at 37 °C and 4 ml aliquots were withdrawn 20 min after exposure to the toxic compounds. Total RNA was extracted using the GenElute Total RNA purification kit (Sigma) following the manufacturer’s instructions. Total RNA was treated with 2 U DNase I to remove trace amounts of DNA. cDNA synthesis was carried out at 37 °C for 1 h in 25 µl of a mixture that contained 2.5 pmol of the specific reverse primers (see below), 10 µl template RNA (5 µg), 0.2 mM dNTPs, 1 µl nuclease-free water, 4 µl 5× buffer [250 mM Tris/HCl pH 8.3, 375 mM KCl, 15 mM MgCl_2_, 10 mM DTT, 40 U RNasin and 200 U M-MLV reverse transcriptase (Invitrogen)]. Relative quantification of transcript levels was performed by qRT-PCR using the Brilliant II SYBR Green QPCR master reagent kit and the Mx3000P detection system (Stratagene). 16S rRNA levels were used for normalization. The qRT-PCR mixture (20 µl) contained 1 µl cDNA template, 120 nM each primer (*sodARTFw* and *sodARTRv* for the *sodA* gene; *sodBRTFw* and *sodBRTRv* for the *sodB* gene; 16SFw and 16SRv for the 16S rRNA gene) and 10 µl ROX reference dye (1 : 200). The qRT-PCR was performed under the following conditions: 10 min at 95 °C followed by 40 cycles of 30 s at 95 °C, 45 s at 53 °C and 30 s at 72 °C, followed by a melting cycle from 53 to 95 °C to check for amplification specificity. A previous standard quantification curve with serial dilutions of RT-PCR products was constructed for each gene to calculate the amplification efficiency. These values were used to obtain the ratio between the gene of interest and the expression of the 16S rRNA gene as described by Pfaffl (2001). All experiments were performed for three biological and technical replicates. The graphics were performed using Graphpad Prism 5 software.

#### Bioinformatic analysis.

Bioinformatic analyses in search of putative BBSs at the *sodA* promoter region were performed using the Vector NTI software and the consensus sequences described by Nishino *et al.* (2005) and Yamamoto *et al.* (2008).

#### Protein purification.

Briefly, *E. coli* BL21(DE3) cells harbouring plasmid pET-TOPO-*baeR* were grown in 500 ml LB medium supplemented with ampicillin (100 µg ml^−1^) to OD_600_ ~0.4 and protein overexpression was carried out by adding 1 mM IPTG and growing for a further 6 h. His-tagged BaeR used in EMSAs was purified as previously described (Guerrero *et al.*, 2012).

#### Construction of transcriptional fusions with the *lacZ* reporter gene.

The native *sodA* promoter region from positions +1 to −600 (with respect to the translation start site) was amplified by PCR with primers pLacZ_SodA_−600Fw and pLacZ_SodA_+1Rv using gDNA from *S*. Typhimurium as a template (strain 14028s). The restriction sites (*Kpn*I and *Hin*dIII, respectively) at the ends of the DNA fragment were introduced by the PCR primers (underlined sequences, Table 2) and digested with the corresponding enzymes. The digested PCR product was cloned into the multiple cloning site of the pLacZ-Basic reporter vector (GenBank accession no. U13184; Clontech) generating the p*sodA*-*lacZ* plasmid. To generate the pMutA-*lacZ* and pMutB-*lacZ* plasmids, the primers used to generate overlapping PCR products spanning the whole length of the *sodA* promoter were: pLacZ_SodA_−600Fw with pSodA_MUTA_Rv or pSodA_MUTB_Rv and pLacZ_SodA_+1Rv with pSodA_MUTA_Fw or pSodA_MUTB_Fw (Table 2). The PCR was performed under the following conditions: 5 min at 95 °C, followed by 10 cycles of 30 s at 94 °C, 30 s at 40 °C and 2 min at 72 °C, followed by 10 cycles of 30 s at 94 °C, 30 s at 45 °C and 2 min at 72 °C, and 20 cycles of 30 s at 94 °C, 30 s at 50 °C and 2 min at 72 °C, and a final extension of 10 min at 72 °C. The resulting PCR products were used as templates in a second reaction with primers pLacZ_SodA_−600Fw and pLacZ_SodA_+1Rv, under the PCR conditions 10 min at 95 °C, followed by 30 cycles of 30 s at 95 °C, 30 s at 55 °C and 1 min at 72 °C, and a final extension of 10 min at 72 °C, to generate the mutated *sodA* promoter, which was digested and cloned into the multiple cloning site of the pLacZ-Basic plasmid to generate pMutA-*lacZ* and pMutB-*lacZ*. Mutations of both sites were generated in the same way, generating the pMutAB-*lacZ* plasmid. Constructions were confirmed by DNA sequencing. The constructs were transformed into the 14028s and Δ*baeSR* strains. To evaluate activity, cells grown at OD_600_ ~0.4 were treated for 30 min with 0.91 µM CIP or 50 µM MEN. Control cells received no treatment. β-Galactosidase activity was determined as described by Miller (1972) and modified by Gil *et al.* (2007).

**Table 2.** Primers used in this study

Primer name	Sequence
*sodARTFw*	5′-TGTGGGAACACGCTTACTACC-3′
*sodARTRv*	5′-CCACGTTCCAGAACTCTTTGA-3′
*sodBRTFw*	5′-TTACATCGACTACCGCAACG-3
*sodBRTRv*	5′-AGTTAACCAGCGCCCAGAA-3′
16SFw	5′-GTAGAATTCCAGGTGTAGCG-3′
16SRv	5′-TTATCACTGGCAGTCTCCTT-3′
pLacZ_SodA_−600Fw*	5′-CGGGGTACCCCGGACGACACTTTAGTGAT-3′
pLacZ_SodA_+1Rv*	5′-CCCAAGCTTAATCATCTCCATTATTGTCG-3′
pSodA_MUTA_Rv†	5′-ACGTATAAAACCAGGTTG**AGGAG**TGATTCCCTCGCAATTGT-3′
pSodA_MUTB_Rv†	5′-ACAATTGCGAGGGAATCA**CTCCT**CAACCTGGTTTTATACGT-3′
pSodA_MUTA_Fw†	5′-TGTGAAATTATAACCTT**AGGAG**TTTTGCCACCGCTGACAA-3′
pSodA_MUTB_Fw†	5′-TTGTCAGCGGTGGCAAAA**CTCCT**AAGGTTATAATTTCACA-3′
*mdtARTFw*	5′-TTCTGGTTCTGGCATAGCCG-3′
*mdtARTRv*	5′-TAACGGTATTCGCGGCGGTC-3′

* Underlined sequences indicate restriction sites for *Kpn*I or *Hin*dIII that were introduced in the primers.

† Bold sequences indicate the mutagenic region introduced in the primers.

#### EMSA.

To study protein–DNA interaction between BaeR and the *sodA* promoter region, non-radioactive EMSAs were performed according to the protocol described by De la Cruz *et al.* (2007) and modified by Gil *et al.* (2009). The probes were obtained by PCR using specific primers pLacZ_SodA_−600Fw and pLacZ_SodA_+1Rv, to amplify the promoter region of *sodA* (600 bp). As a negative control, a 199 bp fragment from the coding region of *mdtA* was used. The fragment was generated using *mdtARTFw* and *mdtARTRv* primers. The PCR was performed under the following conditions: 10 min at 95 °C, followed by 30 cycles of 30 s at 95 °C, 30 s at 55 °C and 1 min at 72 °C, and a final extension of 10 min at 72 °C. DNA fragments with mutations in the BBS boxes A and B were generated by PCR using primers pLacZ_SodA_−600Fw and pLacZ_SodA_+1Rv and plasmids pMutA-*lacZ*, pMutB-*lacZ* and pMutAB-*lacZ* as templates. For the preparation of phosphorylated BaeR (BaeR-P) used in DNA-binding studies, a standard phosphorylation reaction was used in which the protein (60 µg ml^-1^, final concentration) was incubated for 1 h at 30 °C in a buffer containing 100 mM Tris-HCl (pH 7), 10 mM MgCl_2_, 125 mM KCl and 50 mM disodium carbamyl phosphate (Sigma) (Guerrero *et al.*, 2012). Both the promoter region and the negative control (~2 ng ml^−1^) were mixed with increasing amounts of purified BaeR-P in the presence of binding buffer [10 mM Tris-HCl (pH 7.5), 50 mM KCl, 5 mM MgCl_2_ and 2.5 % (v/v) glycerol]. The mixture was incubated for 30 min at room temperature and loaded onto a native 6 % polyacrylamide gel in 0.5× Tris/borate-EDTA buffer. The DNA bands were visualized by ethidium bromide staining on a UV transilluminator. All primers used in this work were designed using the Vector NTI 10 Software.

## Results

### BaeSR TCS modulates SOD activity in response to CIP

**Fig. 1.**
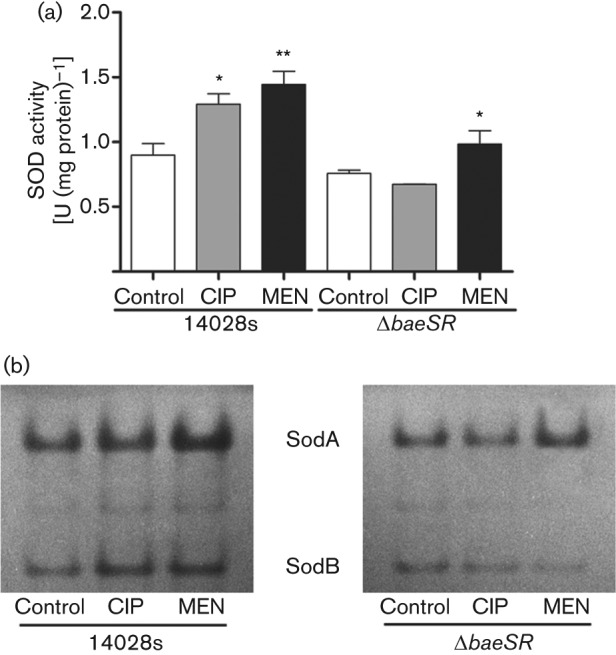
Role of BaeSR in modulating SOD activity in response to CIP. Bacterial cultures were grown to OD_600_ ~0.4 and exposed to CIP (0.91 µM) and MEN (50 µM) for 10 min. Control cells received no treatment. Treatment and strain from which protein were extracted are indicated under the figure. (a) Determination of total SOD activity in crude protein extracts. Unit of SOD activity, 50 % inhibition of NBT reduction. (b) Determination of SOD activity in-gel from the different strains. Names of SOD isozymes as described by Niederhoffer *et al.* (1990) are indicated. Experiments were repeated three times and asterisks represent statistically significant differences as compared with untreated cells from each strain (*P≤0.05; **P≤0.01). Error bars, ±sd.

The BaeSR TCS regulates the expression of *mdtA* in response to CIP (Guerrero *et al.*, 2012), and *S*. Typhimurium Δ*baeSR* and Δ*mdtA* strains are more sensitive to the antibiotic (MIC 3.64 µM for wild-type, 1.82 µM for Δ*baeSR* and Δ*mdtA* strains). We speculated that the role of BaeSR in the response to CIP was not limited to activating efflux systems like *mdtABCD*, but could also be implicated in modulating SOD activity, as suggested by Kaldalu *et al.* (2004) and Smirnova *et al.* (2012). To test this hypothesis, we analysed SOD activity in crude protein extracts from cells treated with CIP or MEN (menadione, superoxide-generating agent as described by Yu, 2007), as described by Beauchamp & Fridovich (1971). In the wild-type strain, total SOD activity increased by 45 and 52 % after CIP and MEN exposure, respectively. Conversely, in the Δ*baeSR* strain total SOD activity was lower after CIP treatment, as compared with untreated cells, while MEN treatment increased the activity, but at levels that were lower than in the wild-type strain exposed to MEN (Fig. 1a). This suggests that BaeSR could modulate total SOD activity. To further investigate the role of BaeSR in this process, and determine if its regulatory effect was over all SOD isozymes or one in specific, we determined SOD activity on native polyacrylamide gels as described elsewhere (Touati, 1983). As shown in Fig. 1(b), in the wild-type strain the major contribution to SOD activity after CIP and MEN treatment corresponds to SodA (Mn-SOD) and SodB (Fe-SOD), which were increased with respect to untreated cells (Fig. 1b). In contrast, in the Δ*baeSR* strain the activity of both enzymes remained almost unaltered after CIP treatment, and only the activity of SodA was slightly increased after MEN treatment, although the levels were significantly lower than in wild-type cells under the same condition (Fig. 1b). Activity of the periplasmic SODs (SodCI/II) was not detected, independent of the genetic background, and therefore was not further investigated. Taken together, our results indicate that the BaeSR TCS modulates SOD activity in response to CIP and MEN, and suggests that the system exerts its effect by regulating the levels of SodA and SodB.

### SodA is upregulated by the BaeSR TCS in response to CIP

**Fig. 2.**
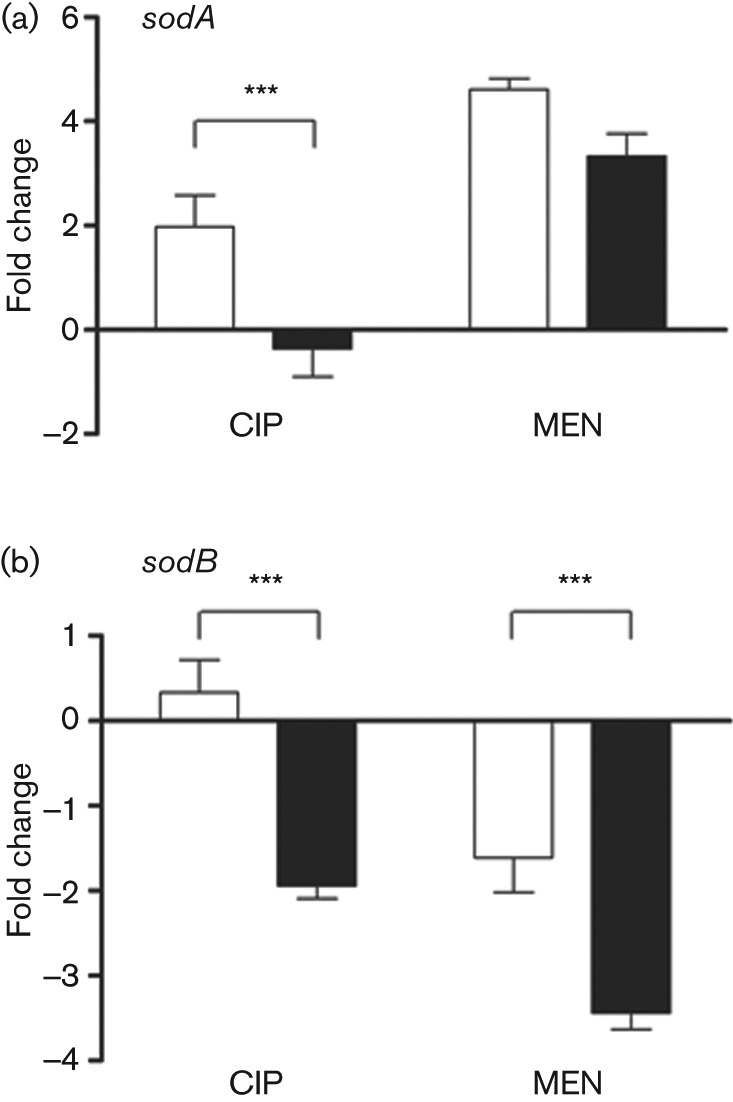
Effect of CIP on the expression of *sodA* and *sodB* in *S*. Typhimurium 14028s and Δ*baeSR*. Exponentially growing cells were exposed to CIP (0.91 µM) and MEN (50 µM) for 10 min and RNA was extracted. Controls received no treatment. The transcript levels of *sodA* (a) and *sodB* (b) were detected by qRT-PCR in strains 14028s (white bars) and Δ*baeSR* (black bars). Experiments were repeated three times and results were normalized using the transcript levels of the 16S rRNA. Asterisks represent statistically significant differences between strains treated with the same toxic compound (***P≤0.001). Error bars, ±sd.

To correlate the changes in SOD activity after CIP and MEN treatment that depended on BaeSR with changes at the transcriptional level, we determined the transcript levels of *sodA* and *sodB* in the different genetic backgrounds. In the wild-type strain, the transcript levels of *sodA* (Fig. 2a) were increased after exposure to CIP and MEN (2- and 4.6-fold change, respectively). In contrast, the transcript levels of *sodB* (Fig. 2b) remained almost unaltered after CIP treatment, while in response to MEN the transcript levels of *sodB* decreased (Fig. 2b). In the *baeSR* mutant, the positive regulation of *sodA* observed in the wild-type strain in response to CIP was abolished (Fig. 2a), while the transcript levels of *sodB* (Fig. 2b) were decreased (−1.94-fold change). In response to MEN, the transcript levels of all of the genes under study retained the regulation observed in the wild-type strain (Fig. 2a, b), indicating that they are differentially expressed in response to both toxic compounds. These results indicate that, in response to CIP, the BaeSR TCS is required to upregulate *sodA* expression and to maintain the levels of *sodB*.

### BaeR binds to the *sodA* promoter region

**Fig. 3.**
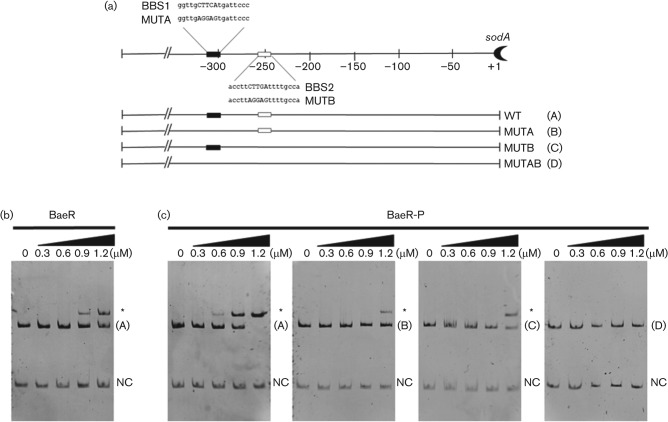
Evaluating BaeR binding at the *sodA* promoter. (a) Schematic representation of the *sodA* promoter region. BaeR boxes BBS1 (black), BBS2 (white), and substitutions generated at the *sodA* promoter (native and substituted bases are in upper case) are shown. The name and sequence of each box are shown. Absence of a rectangle in the scheme represents mutation of the corresponding binding site. A letter (A–D) represents the name of each fragment, as indicated. (b) EMSA using increasing concentrations of BaeR with the wild-type promoter, or (c) EMSA using increasing concentrations of BaeR-P and the fragments (A–D) schematized in (a). NC, negative control. Asterisk indicates DNA–protein interaction. The interactions were resolved by native polyacrylamide gel (6 %) electrophoresis. Bands were visualized by ethidium bromide staining.

To determine if the changes at the transcriptional level were due to a direct interaction of BaeR with the promoter regions of *sodA* and *sodB*, we performed a bioinformatic analysis searching for putative BBSs. As shown in Fig. 3(a), the analysis predicted the presence of two potential binding sites at the promoter of *sodA*, named BBS-1 (GGTTGCTTCATGATTCCC) and BBS-2 (ACCTTCTTGATTTTGCCA). For *sodB*, the analysis did not predict binding sites (data not shown). BBS-1 and BBS-2 showed an identity of 78  and 67 % to the consensus site described by Nishino *et al.* (2005) (TTTTTCTCCATDATTGGC) and 67 and 44 % to the consensus site described by Yamamoto *et al.* (2008) (TCTNCANAA). Alignment of BBS-1 and BBS-2 with BBSs described elsewhere (Nishino *et al.*, 2005; Yamamoto *et al.*, 2008) allowed us to identify a conserved CTNCA element (underlined in the sequence), for which BBS-1 and BBS-2 showed an identity of 100 and 80 %, respectively. To confirm the interactions between BaeR and the predicted sites, we performed EMSAs using a PCR product spanning the promoter region from position −600 to +1 with respect to the translational start site (fragment A, Fig. 3a) and increasing amounts of purified BaeR or BaeR-P. Both BaeR and BaeR-P were able to bind to the wild-type promoter (Fig. 3b, c), although at different concentrations (0.9 and 0.6 µM, respectively), indicating that phosphorylation of BaeR increases its affinity for the promoter of *sodA*. To evaluate the requirement of BBS-1 and BBS-2 for BaeR-P binding *in vitro*, the CTNCA element was mutated individually or with the promoter of *sodA* (Fig. 3a). EMSAs showed that individually mutating BBS-1 or BB2-2 required doubling the amount of BaeR-P to generate a shift in the electrophoretic mobility, as compared with the wild-type promoter (Fig. 3c, fragments B and C, respectively), while mutation of both sites eliminated the interaction even at 1.2 µM BaeR-P (Fig. 3c, fragment D). This indicates that BBS-1 and BBS-2 are required for BaeR-P binding *in vitro*, and suggests that they are required for the observed regulation of *sodA* expression.

### The promoter region of *sodA* has one functional BBS box

**Fig. 4.**
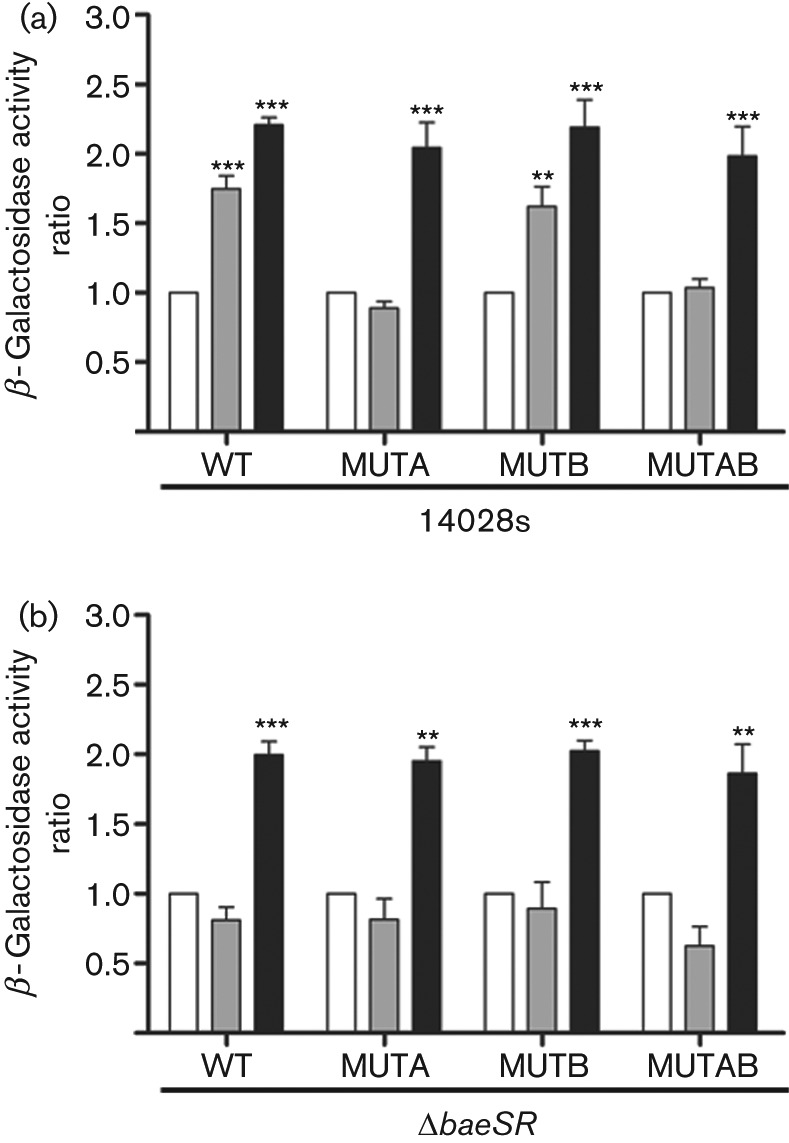
Evaluating the functionality of BBS at the *sodA* promoter. Activity of the wild-type and mutagenized regulatory region of *sodA* in *S*. Typhimurium wild-type (a) and Δ*baeSR* (b) strains. Cells were grown to OD_600_ ~0.4 and treated with CIP (0.91 µM, grey bars) or MEN (50 µM, black bars) and β-galactosidase activity was measured 30 min after treatment. Control cells received no treatment (white bars). Values represent the mean of three independent experiments ±sd (**P≤0.01; ***P≤0.001).

To determine if the BBSs were functional *in vivo*, we constructed transcriptional fusions with the fragments schematized in Fig. 3(a). The different constructions were transformed into strains 14028s and Δ*baeSR*, and β-galactosidase activity was measured after 30 min of CIP or MEN exposure. Wild-type and Δ*baeSR* cells harbouring the native or mutated constructs showed increased activity after MEN treatment (Fig. 4a, b), further demonstrating that the upregulation of *sodA* in response to MEN is independent of the BaeSR TCS, and of BBS-1 or BBS-2. In the wild-type strain with the native promoter or mutated at BBS-2, CIP exposure resulted in increased activity (Fig. 4a). In contrast, when BBS-1 was mutated there were no changes in the activity after CIP exposure, indicating that this regulatory element is required for the positive regulation of *sodA*. In the *baeSR* mutant strain CIP exposure had no effect on β-galactosidase activity, independent of the construct, as compared with untreated cells (Fig. 4b). In sum, the results from EMSAs and transcriptional fusions indicate that BaeR positively regulates *sodA* in response to CIP by a direct interaction with its promoter region, most likely by binding to BBS-1.

## Discussion

It has been proposed that bactericidal antibiotics can induce cellular death through a common mechanism of oxidative damage that relies on the production of ROS, principally O_2_^−^ (Kohanski *et al.*, 2007). Gram-negative bacteria genomes code for enzymes that dismutate O_2_^−^: two cytoplasmic SODs (SodA and SodB) and a periplasmic zinc cofactored isozyme (SodCII) (Korshunov & Imlay, 2002; Fang *et al.*, 1999). In *S*. Typhimurium, a second periplasmic SOD, denominated SodCI, is also present (Fang *et al.*, 1999; Figueroa-Bossi & Bossi, 1999).

Based on the model of antibiotic killing through ROS production (Kohanski *et al.*, 2007), and the fact that the BaeSR TCS participates in the response to CIP (Guerrero *et al.*, 2012), we speculated that BaeSR could contribute to CIP resistance by upregulating the transcript levels of the genes that code for SODs. In agreement, total SOD activity was increased in the wild-type strain exposed to CIP, and was dependent on BaeSR (Fig. 1). Additionally, *sodA* transcript levels were increased in response to CIP, while the levels of *sodB* remained unaltered; however, in the *baeSR* mutant the levels of *sodA* and *sodB* were decreased after exposure to the antibiotic (Fig. 2).

During the course of this study, two reports showed that killing by bactericidal antibiotics was not mediated by ROS production (Keren *et al.*, 2013; Liu & Imlay, 2013). However, Keren *et al.* (2013) showed evidence that at least for norfloxacin, a quinolone with a similar mechanism of action to CIP (Drlica & Zhao, 1997), the MIC of the antibiotic under aerobic conditions was half that of anaerobically grown *E. coli* cells, and killing was higher under aerobic conditions when 0.125 and 0.25 µg ml^−1^ of norfloxacin was used. These results suggest that at certain antibiotic and oxygen concentrations ROS could be generated, although this discrepancy was not addressed and further studies are required to confirm or rule out this possibility. Despite the recent studies of antibiotic ROS-independent killing mechanisms, there is evidence that SOD expression is increased in response to antibiotics. Expression of *sodA* and *sodB* is induced by ofloxacin (Kaldalu *et al.*, 2004), and *sodA* also by CIP (Smirnova *et al.*, 2012), in agreement with findings from this study. Whether this increase in expression is a consequence of ROS production derived from CIP treatment remains to be determined, as well as the physiological role of SOD in response to CIP, if the mechanism is independent of ROS production.

In the present study, we focused on the role of BaeSR in regulating SOD expression in response to CIP by using sublethal concentrations of the antibiotic, and demonstrated that under this condition *sodA* is activated when CIP is amended. Additionally, BaeSR may contribute to CIP resistance by maintaining the basal levels of *sodB* (Fig. 2b). At the concentration of CIP studied, the role of BaeSR could be to detect the antibiotic and as a consequence activate detoxifying machinery, like Mn-SOD (*sodA*) (Kaldalu *et al.*, 2004), and/or activate transcription of the *mdtABCD* operon to promote antibiotic efflux (Nishino *et al.*, 2007). Since BaeSR is implicated in metal resistance (Nishino *et al.*, 2007), and CIP may bind Mg^2+^ (Lindner *et al.*, 2002), one possibility is that BaeS detects CIP by the recognition of this metal.

Interestingly, neither *sodA* nor *sodB* mutant strain is sensitive to CIP (Goswami *et al.*, 2006). It is possible that the role of the different isozymes could be partially redundant, explaining why individual mutations of either *sodA* or *sodB* do not increase the sensitivity. However, in the *baeSR* mutant strain treated with CIP, downregulation of *sodB* and loss of upregulation of *sodA* (Fig. 3) could lead to an increase in oxidative stress, among other conditions, leading to the sensitivity. A similar phenomenon was observed for the genes involved in H_2_O_2_ degradation. Individual *katE*, *katG* and *ahpCF* mutant strains do not present increased sensitivity towards CIP; however, double and triple mutants are highly sensitized, supporting the notion that their responses partially overlap and are required for CIP resistance (Goswami *et al.*, 2006).

The transcription factor SoxS positively regulates *sodA* in response to paraquat (Pomposiello *et al.*, 2001); however, our analysis shows that SoxS does not contribute to *sodA* regulation in response to CIP (data not shown). The fact that two transcription factors positively regulate *sodA* in response to ROS, or possibly in response to ROS generated by exposure to antibiotics, could be explained by a model in which they respond to different concentrations of O_2_^−^. Supporting this hypothesis, Martin *et al.* (2008) demonstrated that SoxS is activated at high concentrations of O_2_^−^, while CIP is suggested to generate low levels of O_2_^−^ (Goswami *et al.*, 2006), and therefore *sodA* upregulation by SoxS and/or BaeR could be dose dependent. Our results indicate that BaeR modulates the expression of *sodB* (Fig. 2b, 3b); however, EMSAs suggest that its role is indirect (data not shown). It has been reported that expression of *sodB* is regulated at the transcriptional and post-transcriptional level by several factors including Fur, NsrR and the sRNAs *ryhB* and *fnrS* (Massé & Gottesman, 2002; Durand & Storz, 2010; Niederhoffer *et al.*, 1990; Partridge *et al.*, 2009). One possibility is that BaeR could modulate the levels of one or more of these factors or sRNA, and in this manner regulate *sodB*, although this requires further investigation.

In *E. coli* the BaeSR TCS has been related to drug (Baranova & Nikaido, 2002) and metal resistance by regulating the expression of genes coding for drug efflux systems (Hu *et al.*, 2005; Yamamoto *et al.*, 2008). Moreover, in *E*. *coli* BaeSR has been related to envelope stress response (Leblanc *et al.*, 2011) and zinc toxicity response (Wang & Fierke, 2013) and activates *spy* in association with CpxARP (Rosner & Martin, 2013). In *S*. Typhimurium BaeSR has been related to multidrug and metal resistance (Nishino *et al.*, 2007), ceftriaxone resistance (Hu *et al*., 2005) and tungstate waste disposal (Appia-Ayme *et al.*, 2011). Our results indicate that, in addition to the aforementioned, the BaeSR TCS plays a role in the response to CIP by regulating the expression of SodA and possibly of SodB. To our knowledge, this is the first evidence relating this TCS system with this regulation, suggesting that its role in CIP resistance could be more complex. Further studies to determine the genes regulated by BaeSR in response to CIP, the molecular mechanism of BaeSR activation by CIP, and if CIP treatment generates ROS are required, and are being conducted in our laboratory.
